# Meckel's diverticulum as a lead point for intussusception in the first day of life: A case report of neonatal perforation

**DOI:** 10.1016/j.ijscr.2025.111548

**Published:** 2025-06-19

**Authors:** Usama Qumsieh, Alaa R. Al-Ihribat, Shahd B. Shawar, Ahmed J.M. Doudin, Omar A. Alhroub, Omar Y.A. Hajjaj

**Affiliations:** a Palestine Polytechnic University, Faculty of Medicine, Hebron, Palestine; b Pediatric Surgery Department, Al-Ahli Hospital, Hebron, Palestine; c General Surgery Department, Al-Ahli Hospital, Hebron, Palestine; d Al-Quds University, Faculty of Medicine, Abo-Dis, Palestine

**Keywords:** Neonatal intussusception, Meckel's diverticulum, Bowel perforation, Term neonate

## Abstract

**Introduction and importance:**

Intussusception in neonates is extremely rare, especially in full-term newborns. Even rarer is the occurrence of Meckel's diverticulum (MD) acting as a lead point within the first 24 h of life. Prompt recognition is critical as delays can lead to bowel perforation and life-threatening complications.

**Case presentation:**

We report a case of a 13-hour-old full-term female neonate who presented with abdominal distension and signs of sepsis shortly after her first feed. Imaging revealed pneumoperitoneum, prompting emergent laparotomy. Intraoperatively, ileoileal intussusception with bowel perforation was identified, with Meckel's diverticulum acting as the lead point. The affected segment was resected with primary anastomosis. The patient required cardiopulmonary resuscitation intraoperatively but eventually made a full recovery after a second-look surgery and NICU support.

**Discussion:**

This case represents one of the earliest presentations of MD-related intussusception complicated by bowel perforation in a term neonate. Diagnosing intussusception in neonates is challenging due to atypical presentation. Although MD is the most common congenital GI anomaly, it rarely presents symptomatically in neonates, let alone as a cause of intussusception and perforation on day one of life.

**Conclusion:**

Neonatal intussusception, particularly in term infants, should not be overlooked in the differential diagnosis of early abdominal emergencies. Meckel's diverticulum, despite its rarity as a lead point in this age group, must be considered to avoid delayed diagnosis and serious complications.

## Highlights

• Neonatal intussusception is a rare cause of bowel obstruction in full-term neonates.
• Meckel’s diverticulum may act as a lead point for intussusception in the first 24 hours.
• Abdominal distension and pneumoperitoneum needs urgent surgical evaluation in neonates.
• Consider intussusception and Meckel’s diverticulum in neonatal abdominal emergency diagnosis.

## 1 Introduction

Intussusception is a common clinical entity with well-defined features in later infancy and young childhood. It typically presents between 6 and 18 months of age [1]. However, Neonatal intussusception, which is seen in the first month of life, is extremely rare, with an incidence of less than 1 % of all cases of intussusception. [2].

Intussusception classically presents with one or all of these tetrad clinical features: sudden onset of colicky abdominal pain, bilious vomiting, bloody stools, and a palpable abdominal mass. However, Neonatal intussusception may be difficult to recognize as a result of its rarity and its confusing clinical picture, as classic symptoms such as abdominal pain and a palpable mass are uncommon [3].

The etiology remains unknown in the majority of cases of intussusception. In neonates, a lead point lesion such as a duplication cyst, hamartoma, Meckel's diverticulum, or mesenchymoma was reported [4].

Meckel's diverticulum, a remnant of the vitelline duct, is the most common congenital gastrointestinal anomaly, occurring in approximately 2 % of the population. However, it's rarely symptomatic in neonates. Complications such as perforation, segmental ileal dilatation, volvulus or, rarely, intussusception, are possible [5].

We report a very rare case of neonatal intussusception in a full-term neonate secondary to Meckel's diverticulum, which was complicated by bowel perforation in the first 24 h of life. Aiming to highlight the importance of considering this clinical entity, as subsequent complications might be devastating.

This case report adheres to the SCARE 2025 guidelines for surgical case reporting [14].

## 2 Case presentation

A 13-hour-old newborn female, with a gestational age of 39 weeks, was born by urgent CS due to fetal distress and thick meconium. Her Apgar scores were 7 and 9 at 1 and 5 min respectively. Birth weight was 2.6 kg. At the age of 5 h following her first breastfeeding, patient started to develop abdominal distention, with no history of vomiting or bleeding per rectum, referred to our hospital for further evaluation.

On arrival, the patient was noted to be hypoactive and dehydrated, her vital signs were as follows: Temperature: 35.6 Rectally, heart rate: 130 bpm, blood pressure 72/37 mmHg (mean arterial pressure 47), respiratory rate: 50 breaths per minute, and oxygen saturation was 100 %. Her abdomen was distended, hyper-resonance to percussion, with tenderness all over her abdomen along with bluish discoloration. The anus was patent and umbilicus had three vessels.

Physical examination revealed no dysmorphic features, good air entry bilateral with no added sounds, regular heart rate with grade I murmur.

Laboratory results showed hemoglobin 20.8, white blood cell count 12.3, platelets count 108, and C-reactive protein 49.

**Fig. 1:**
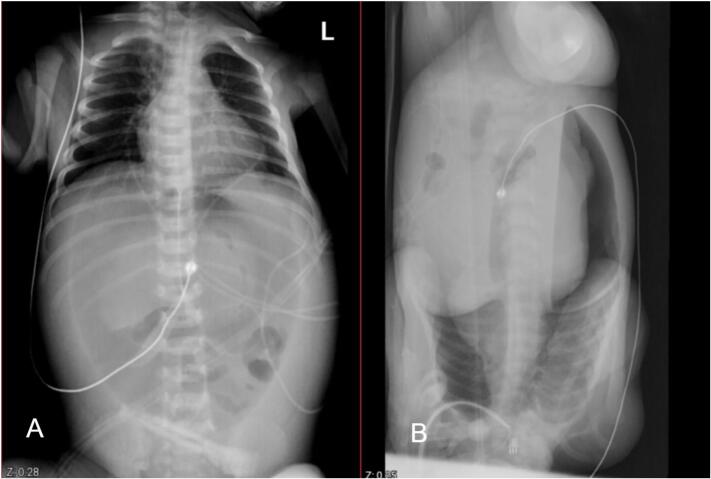
A: Plain abdominal X-ray showing football sign. B: Lateral decubitus abdominal X-ray demonstrating pneumoperitoneum.

Supine abdominal and lateral decubitus cross table X-rays demonstrated football sign and pneumoperitoneum, respectively [Fig. 1].

**Fig. 2:**
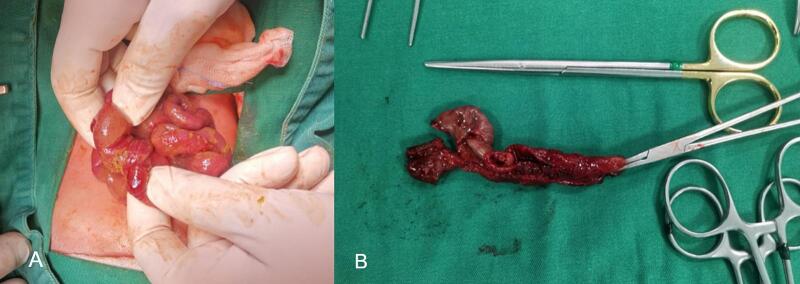
Intraoperative findings: A: ileoileal intussusception complicated by perforation. B: Perforation and leading point (Meckel's diverticulum) in the resected small bowel segment.

Resuscitation with IV-Fluids and Antibiotics was started, then the patient transferred to operating theatre emergently. A right upper transverse exploratory laparotomy was performed. Upon opening the peritoneum, moderate amounts of small bowel content came out, suction and irrigation done. Ileo-ileal intussusception with bowel perforation was identified [Fig. 2], the affected segment resected and primary anastomosis was performed. The leading point was Meckel's diverticulum [Fig. 2]. During the procedure, the patient experienced sudden bradycardia then cardiac arrest, Cardiopulmonary resuscitation (CRP) started, and fortunately, the pulse was restored after one cycle. The skin closed via stapler for second look. The patient transferred to neonatal intensive care unit (NICU), intubated and continued on intravenous fluids, antibiotics and circulatory support.

Over the next 36 h the patient showed gradual improvement. A second look laparotomy was performed and the abdomen was found in a good condition, closed in layers. The patient was extubated few days later and continued to improve clinically and laboratory.

**Fig. 3:**
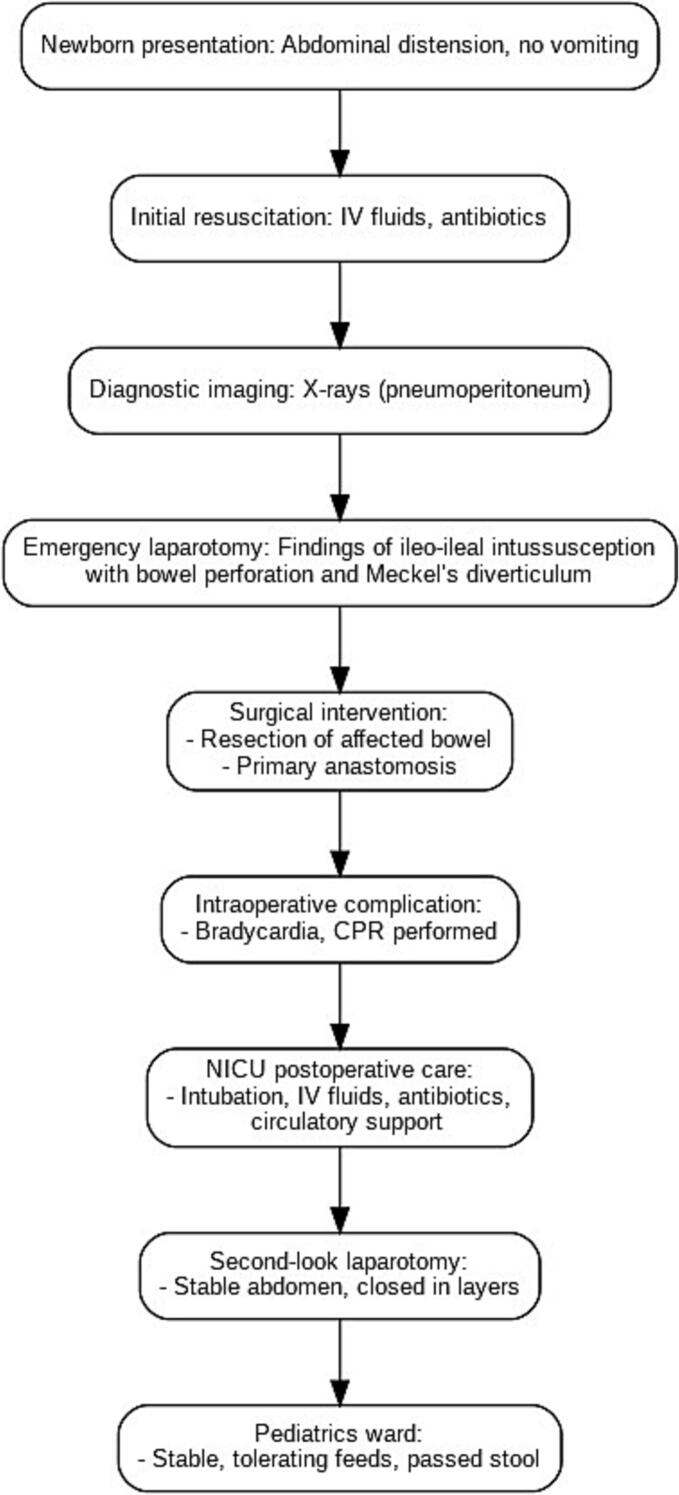
Management flowchart.

Pathology results confirmed the presence of Meckel's diverticulum. The patient transferred to pediatrics ward few days later, with stable vital signs, tolerated diet and passed stool. A management flowchart for this baby is shown in Fig. 3.

## 3 Discussion

Intussusception occurs when a segment of the bowel telescopes into an adjacent bowel segment, leading to complications such as obstruction or intestinal ischemia. This condition is more common in children and presents with the classic triad of cramping abdominal pain, bloody diarrhea and a palpable tender mass [6]. Neonatal intussusception is uncommon, with most cases diagnosed postnatally in preterm infants, often linked to necrotizing enterocolitis (NEC) [7]. Among all cases of neonatal intestinal obstruction, only 3 % are caused by intussusception, in a 150-year review of the world's literature by Rachelson et al. in 1955, the average incidence of intussusception in the first month of life was 0.3 % of all cases of intussusception [2].

The etiology of intussusception in children is typically idiopathic, often influenced by anatomic or infectious factors [8]. Avansino et al. reported lead point lesions in 10–58 % of cases in newborns [9]. The reported causes in literature are duplication cyst, hamartoma, Meckel's diverticulum and mesenchymoma [3]. In full term babies, Cases of congenital infantile fibrosarcoma [10], jejunal atresia [3], cecal duplication cyst have been reported to cause intestinal intussusception. However, Intussusception due to MD is exceedingly rare in term neonates, making our case an exceptional occurrence.

Although MD is the most common congenital GI anomaly, its symptomatic presentation in neonates is rare. In a report by Yeh et al. [12], a full-term neonate developed MD perforation with pneumoperitoneum at approximately 24 h of life. Liaqat et al. [15] reviewed six cases of neonatal MD perforation, most presenting within the first month of life—but none had associated intussusception. To the best of our knowledge, our case represents the earliest-reported MD-related ileoileal intussusception with perforation in a term neonate within the first 24 h of life.

Intussusception occurring in the neonate is still difficult to assess and diagnosing MD and intussusception in neonates is challenging due to the nonspecific presentation, including abdominal distension, feeding intolerance, and sepsis-like symptoms. Radiologic findings such as pneumoperitoneum or a “target sign” on ultrasound may indicate complications but are often nonspecific in neonates. In our case, initial abdominal X-rays showed the football sign and pneumoperitoneum, suggestive of intestinal perforation. Early surgical exploration remains the gold standard for definitive diagnosis in critically ill neonates [11]. These findings emphasize that in any full-term neonate with early signs of abdominal sepsis and pneumoperitoneum, MD should be considered in the differential—especially when imaging reveals features suggestive of obstruction or perforation.

Our case involves a 13-hour-old term neonate who developed abdominal distension shortly after the first feed. Upon exploration, an ileoileal intussusception with MD as the lead point and associated bowel perforation was identified. Although cases of neonatal MD with complications have been reported, they differ from our case in various aspects, Kumar et al. [12] described a full-term neonate with MD-associated perforation, but there was no intussusception component, additionally, Hassan et al. [13] presented a case of neonatal intussusception with perforation, but the patient was diagnosed at three weeks of life and had a colonic involvement rather than an ileoileal type. These cases highlight the rarity of our case as MD-related ileoileal intussusception complicated by bowel perforation within the first 24 h of life.

The management of neonatal MD complicated by intussusception and perforation is complex. Prompt resuscitation with intravenous fluids, antibiotics, and urgent surgical intervention is critical. In our case, the patient required extensive resuscitation, an emergent laparotomy, and a second-look surgery due to the severity of the initial presentation. The sudden intraoperative cardiac arrest further complicated the course, requiring immediate cardiopulmonary resuscitation. Despite these challenges, the patient showed remarkable recovery, emphasizing the importance of early recognition and multidisciplinary neonatal surgical care.

## 4 Conclusion

Neonatal intussusception is a rare entity appearing mainly in preterm infants. In full-term babies, this abdominal emergency is usually due to an organic lesion. This case is underscoring the need to consider intussusception and Meckel's diverticulum as differential diagnoses even in term neonates. The overall prognosis for neonates with intussusceptions depends on early diagnosis, because once a critical condition develops, as in this case, the mortality rate is likely to rise.

## Author contribution

**Alaa R. Al-Ihribat, Shahd B. Shawar**: Conceptualization, case analysis, manuscript writing, and editing.

**Ahmed J. M. Doudin, Omar A. Alhroub, Omar Y. A. Hajjaj**: Data collection, literature review, and manuscript drafting.

**Usama Qumsieh, Ahmed J. M. Doudin**: Clinical management of the patient, data interpretation, and manuscript revision.

All authors have read and approved the final manuscript and agree to be accountable for all aspects of the work.

## Informed consent

Written informed consent was obtained from the patient's family for publication of this case report and any accompanying images. A copy of the written consent is available for review by the Editor of this journal if requested.

## Ethical approval

Ethical approval was not required for this study according to our institutional policies, which exempt single case reports from Institutional Review Board (IRB) review. However, we ensured full protection of patient privacy and removed any potentially identifiable information.

## Guarantor

Alaa R. AL-Ihribat

## Funding

This research received no specific grant from any funding agency in the public, commercial, or not for-profit sectors.

## Conflict of interest statement

The authors have no conflict of interest to declare.

## Data Availability

The data used to support the findings of this study are included in the article.
